# Nanoplastics in Depolymerization Products from Hydrolysis of Poly(Ethylene Terephthalate) in the Solid State

**DOI:** 10.1002/marc.202500776

**Published:** 2026-02-06

**Authors:** Sierra F. Yost, Peter M. Guirguis, Patricia Pereira, Philip E. Savage, Bryan D. Vogt

**Affiliations:** ^1^ Robert V. Waltemeyer Department of Chemical Engineering The Pennsylvania State University Pennsylvania USA

**Keywords:** chemical recycling, microplastic, nanoparticles

## Abstract

Circularity in plastic waste management through depolymerization to monomer is a promising route to address inefficiencies in the current recycling ecosystem, especially for poly(ethylene terephthalate) (PET). Hydrolysis provides a green solvent to promote the depolymerization of PET, but lifecycle analyses have driven extensive efforts to lower the reaction temperature to minimize greenhouse gas emissions. However, recent work (*Nat. Commun*., 2025, 16, 3051) has indicated that hydrolysis at temperatures near the normal boiling point of water can generate nanoplastics. Here, we demonstrate that PET hydrolysis at temperatures less than 180°C leads to nanoplastics as determined from dynamic light scattering (DLS) and differential scanning calorimetry (DSC). Clear evidence of plastic nanoparticles is observed after hydrolysis at 150°C for three of the four PET sources examined. DSC thermograms of hydrolysis products on heating exhibit a broad peak with a depressed melting point that is consistent with small PET crystals. Hydrolysis at higher temperatures leads to smaller particles with DSC thermograms that are indicative of small molecules and oligomers. These results illustrate the potential for unintended consequences from efforts to reduce GHG emissions with chemical recycling to generate nanoplastics in the product stream that may be difficult to readily differentiate from expected products.

## Graphical abstract

Hydrolysis of PET in the solid generates nanoparticles, but thermal signatures of PET were only observed for hydrolysis at 150°C, while oligomers appeared to dominate the non‐monomeric products from hydrolysis at higher temperatures.

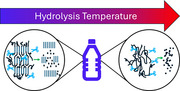

## 1 Introduction

Plastic waste has emerged as a critical environmental challenge in the 21^st^ century as plastic production continues to grow exponentially, with most plastic used in non‐durable goods [1, 2, 3]. Despite significant efforts to promote recycling and enhance the utilization of plastic waste as a resource [4], recycling rates for plastics remain low without significant gains in the past decade [5, 6]. Several issues limit the efficacy of traditional mechanical recycling, including thermo‐oxidative and shear degradation of the polymer during reprocessing [7], limitations in effective sorting of plastic waste [8], and antagonistic additive interactions [9]. As a result of these and other challenges, there has been growing interest in chemical (or advanced) recycling where the plastics are deconstructed to its monomers [10] or high‐value chemicals [11]. These reactive processes require additional energy for reactions and separations, but lifecycle analyses (LCA) indicate potential environmental benefits for chemical recycling processes [12, 13, 14]. The exact pathways available for these transformations are polymer dependent [15, 16]; polymerization of vinyl and olefin monomers results in resilient backbones that are challenging to convert to monomer due to side reactions prior to the ceiling temperature [17]. Conversely, many step‐growth (condensation) polymers can readily be converted back to monomer through solvolysis [18, 19, 20]. The ability to directly convert plastic back to its functional building blocks with high atom efficiency provides a simple route to true circularity [21, 22]. Polyethylene terephthalate (PET) is the most produced and used step‐growth polymer; PET comprises approximately 10% of the total plastic produced annually [1]. PET is also the most readily recycled plastic through mechanical means, but a significant fraction is downcycled from bottle‐grade PET [23]. With the demand for increased recycled content in PET bottles [24], chemical upcycling of waste textile PET to monomer is being commercialized [25] with glycolysis and ethanolysis being the primary solvolysis routes.

However, glycol and ethanol used in these processes are typically petroleum‐based. Replacing these chemicals represents an opportunity to reduce the environmental footprint for chemical recycling of PET. Although biobased ethanol has reduced environmental impacts, its production has a high water use, with between 8 and 160 gallons of water required for one gallon of corn‐derived ethanol [26]. Hydrolysis allows significantly lower water use, even when considering deionization of water [27] and offers a potentially greener approach to depolymerize PET to generate its monomers, terephthalic acid (TPA) and ethylene glycol (EG), as well as bis(2‐hydroxyethyl) terephthalate (BHET), and mono(2‐hydroxyethyl) terephthalate (MHET) [28], although high reaction temperature can lead to other by‐products [29]. The yield of TPA from hydrolysis is limited by equilibrium with co‐products [30]. Additionally, the hydrolysis of PET can be kinetically modulated with pH or other catalysts [31]. To minimize energy consumption in hydrolysis [14], and reduce the degradation of TPA [32], lower temperatures are desired [33]. However, there appears to be a reduction in the yield of TPA from hydrolysis at temperatures below the melting point (T_m_) of PET [20, 28]. This reduction may be associated with reduced accessibility of water to the crystalline phase of PET.

Depolymerization reactions to chemically recycle PET are analogous to some reactions commonly associated with weathering and environmental exposure that occur with mismanaged plastic waste [34]. Recent examination of PET with controlled hydrolysis demonstrated the release of crystalline domains of PET to produce microplastics (MPs) and nanoplastics (NPs) [35], which are defined as polymer particles less than 5 mm and less than 1 µm, respectively [36]. A variety of forces and environmental factors give rise to secondary MPs, which comprise a major source of MPs and NPs in the environment [37, 38]. These plastic particles are ubiquitous [5] and have been found in water, air, plants, and animals, including humans [5, 6, 39, 40, 41, 42]. There is growing evidence that MPs and NPs pose significant health risks [43, 44, 45, 46, 47]. so routes to minimize their generation and release to the environment are important. However, MPs and NPs are easy to generate with reports of MPs from a variety of everyday operations [48], including opening sealed plastic packaging [49] and washing of synthetic textiles [50]. Most reports regarding the generation of MPs occur at near ambient temperatures, with the formation driven from long term environmental exposures and/or mechanical forces.

For plastic recycling through solvolysis, reducing environmental impact favours a reduction in process temperature [14]. With the demonstrated ability of hydrolysis to generate MPs and NPs near the normal boiling point of water [35], there is potential to inadvertently produce MPs and NPs in chemical recycling product streams through operating at lower temperatures; these lower temperatures would be considered to be favourable based on reduced greenhouse gas (GHG) emissions, but these analyses do not tend to consider environmental impact of by‐products from the recycling process. Here, we examine how reaction temperature impacts the generation of NPs in the hydrolysis product stream using a variety of PET sources, including post‐consumer PET. The low solubility of TPA in water leads to challenges in identifying microplastics, as the hydrolysis product is cloudy with TPA dispersed in water. To clarify the solution, dimethylsulfoxide (DMSO), a good solvent for TPA, but not for PET at room temperature, was added to the hydrolysis products. DMSO enables quantification of the solution for PET MPs and NPs through selective dissolution of the TPA product. However, even at 90:10 DMSO to water, the TPA is not fully dissolved, with 200 nm particles determined from dynamic light scattering (DLS). Much larger particles with hydrodynamic radii approaching 1 µm remain in the product stream after 36 h of hydrolysis at 150°C. which are attributed to MPs. Thermal analysis of the dried product stream confirmed the presence of PET from its melting transition that is distinct from TPA and known small molecule products. At higher temperatures for hydrolysis, particles with similar sizes to TPA alone in solution are determined from DLS. However, hydrolysis at 180°C leads to thermograms that are similar the products from hydrolysis at 150°C. These results suggest some PET microplastics may be formed from hydrolysis at 180°C and below. Products from hydrolysis at higher temperatures exhibit sharper thermal transitions in the DSC thermograms that are consistent with BHET and its dimers. This work provides insight into the formation of nanoplastics during chemical recycling at low temperatures and should be cautionary to efforts to reduce depolymerization temperatures significantly below the melting point without characterization for particles. Conversely, these results also illustrate that the formation of particles in solution from solvolysis does not necessarily indicate that microplastics were generated.

## 2 Results and Discussion

**FIGURE 1:**
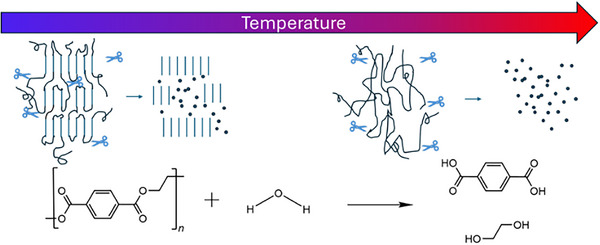
Schematic illustration of hypothesized differences in the PET hydrolysis associated with semicrystalline PET at low temperature and the PET melt at high temperature. The amorphous regions can be effectively broken down to monomers, while the lack of water in the crystalline regions prohibits hydrolysis.

The hydrolysis of PET to its monomers, TPA and EG, has been extensively studied [28], but reaction temperature impacts the reaction through the mobility of the PET segments. At temperatures greater than the melting point of PET, which can be as high as 289°C [51], the reaction occurs between water and the amorphous PET melt. However, at lower temperatures, the PET may remain semi‐crystalline to alter the local solubility of water within the polymer phase, where water is effectively excluded from the crystals [52]. Hydrolysis reactions within the amorphous regions selectively break tie chains connecting the crystals, which leads to loss of mechanical performance under environmental aging [53]. This mechanism of selective tie chain scission has been shown to facilitate the generation of microplastics [35]. Figure 1 schematically illustrates these mechanistic differences to demonstrate the potential to generate microplastics at low hydrolysis (or solvolysis) temperatures. As the melting point depends on the crystal size due to the added surface energy of small crystals [54] and the solubility of water within the amorphous PET will act to depress the melting temperature [55], the temperature at which the crystalline domains will not be hydrolyzed is not obvious.

**FIGURE 2:**
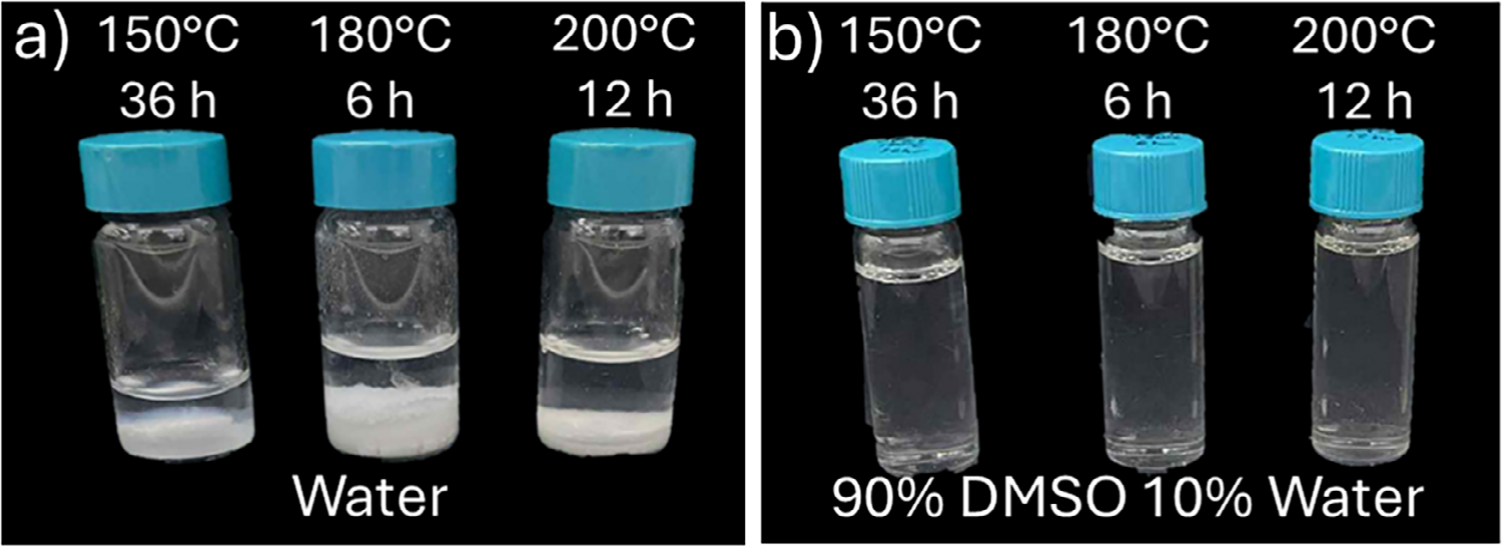
Photographs of the a) products from hydrolysis of ReCryst_vPET at temperatures from 150°C to 200°C where phase separation of the TPA product is observed. Dilution in DMSO to 9:1 (v:v) DMSO: water results in b) clear solution for the same hydrolysis products.

Hydrolysis of PET results in a phase‐separated mixture with an aqueous phase that contains EG, while TPA crystallizes out of solution. Based on our hypothesized mechanism for potential microplastic generation, a higher crystallinity in the PET should lead to more microplastics generated. Figure S1 illustrates the DSC thermograms associated with the four PET specimens examined. A virgin PET (vPET) sheet and the same PET after slow recrystallization (ReCryst_vPET) to maximize crystallinity were used for comparison in the hydrolysis. Additionally, we examined two different post‐consumer PET samples from Diet Coke bottles: a 16.9 oz bottle without recycled content labeling (PC_PET) and a 20 oz bottle labeled as 100% recycled material (PC_rPET). As quantified in Table 1, the highest crystallinity was found for ReCryst_vPET and this specimen was used to understand the hydrolysis conditions at which PET MPs and NPs are formed. Hydrolysis of ReCryst_vPET illustrates no visual difference in the products between reactions performed at 150°C and 200°C as shown in Figure 2. The products from the reactor phase separate with the TPA crystallizing and sedimenting to the bottom of the vials as shown in Figure 2a. There is no apparent difference in the products at the different temperatures. However, the sedimentation of the TPA crystals challenges the identification of any PET microplastics. As TPA dissolves in DMSO [56], addition of DMSO at nine times the volume of the water led to visually clear solutions (Figure 2b). There is some Tyndall effect in the solution from the hydrolysis products, which suggests the presence of particles within the solution.

**TABLE 1:** Thermal transitions and crystallinity of the PET samples.

Sample	T_g_ (°C)	T_c_ / T_m_ (°C)	X_c_ (%)
PC_rPET	82.2	179.4/248.1	30.3
PC_PET	81.8	168.9/246.9	16.8
vPET	73.0	173.5/246.3	12.0
ReCryst_vPET	79.3	204.4/247.7	41.9

**FIGURE 3:**
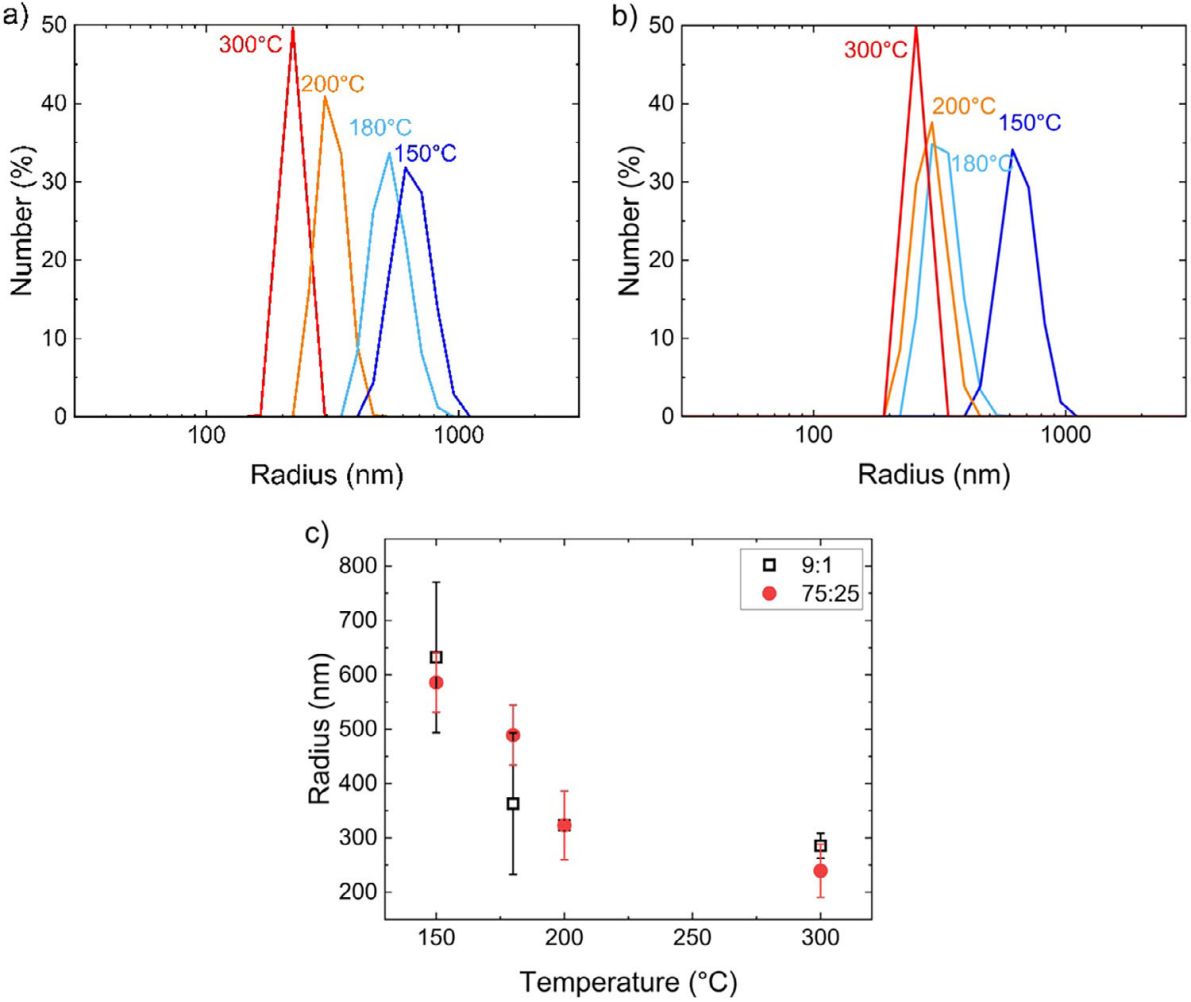
Hydrodynamic radii of hydrolysis products from ReCryst_vPET when dissolved in a) 75:25 and b) 9:1 (v:v) DMSO: water. The hydrolysis temperature is labelled for each of the size distributions. c) Average particle size as a function of the hydrolysis temperature from DLS analysis in (●) 75:25 and (□) 9:1 (v:v) DMSO: water.

Dynamic light scattering (DLS) was used to quantify the size of the particles within these DMSO: water solutions as shown in Figure 3. Multiple measurements were performed for each reaction condition. Figure S2 illustrates DLS data obtained from the hydrolysis of the ReCryst_vPET. The particle size distributions were determined from fits of the autocorrelation function from DLS (Figure S3). At 75:25 (v:v) DMSO:water, the solutions appear transparent and thus appropriate for DLS to quantify for microplastics. From DLS analysis of these 75:25 DMSO: water solutions, there is a decrease in the size of the particles as the reaction temperature is increased (Figure 3a). Reaction at 150 and 180°C leads to large particles between 350 nm and approximately 1 µm. There is appreciable overlap in the particle distributions at these reaction conditions, but the largest particles are obtained from the lowest hydrolysis temperature examined. Hydrolysis at higher temperatures (200 and 300°C) leads to smaller particle sizes, but the data suggest approximately 200 nm particles in the solution from the 300°C hydrolysis. At 300°C, the PET crystals are fully melted and thus should not generate NPs based on the mechanism proposed by Kumar and coworkers [35].

To understand the origins of the light scattering signal from the hydrolysis at 300°C, pure water and DMSO: water solutions were measured; the scattering is near the detection limits, so the particles observed are associated with the PET and its hydrolysis products. However, self‐assembly of TPA on surfaces has been reported to be modulated by details of intermolecular hydrogen bonding [55], so the TPA could potentially be involved with the particles determined from DLS. Figure S4 illustrates the DLS for TPA dissolved in DMSO: water solutions. The particle size for the TPA at 0.075 mg/mL, which is similar to expectation for the hydrolysis products, was measured to be 105 ± 16 nm in 75:25 DMSO: water, and 193 ± 29 nm in a 9:1 DMSO: water. These results suggest that the TPA in these DMSO: water solutions aggregates into approximately 100–200 nm particles. To distinguish assembled TPA structures from PET NPs, the concentration of DMSO was increased to 9:1 (v:v) DMSO: water. Figure 3b illustrates the particle size distributions from DLS for the same hydrolysis products after a change in dilution. There is a significant shift to smaller sizes for the hydrolysis products from 180°C when further diluting with DMSO. This solution concentration dependence of the particle size is inconsistent with PET particles as DMSO is not expected to significantly impact the crystalline PET at room temperature [57]. Thus, the particles in the hydrolysis product from reaction at 180°C are suspected to be comprised primarily of small molecule hydrolysis products and potentially some fraction of PET particles. After dilution, the particle size from the hydrolysis product obtained at 180°C is similar to that observed with higher hydrolysis reaction temperatures, as shown in Figure 3c. However, there is limited change in the particle size for the products from the lowest hydrolysis temperature on dilution from 75:25 to 9:1 DMSO:water. The particle size for the lowest hydrolysis temperature (150°C) is significantly larger than the others. This, along with the relative invariance in the particle size indicate that the products from hydrolysis at 150°C contain a significant fraction of microplastics. The size of the particles at 9:1 dilution is similar for products from hydrolysis at 180, 200, and 300°C, which could be associated with similar small molecule products in solution.

**FIGURE 4:**
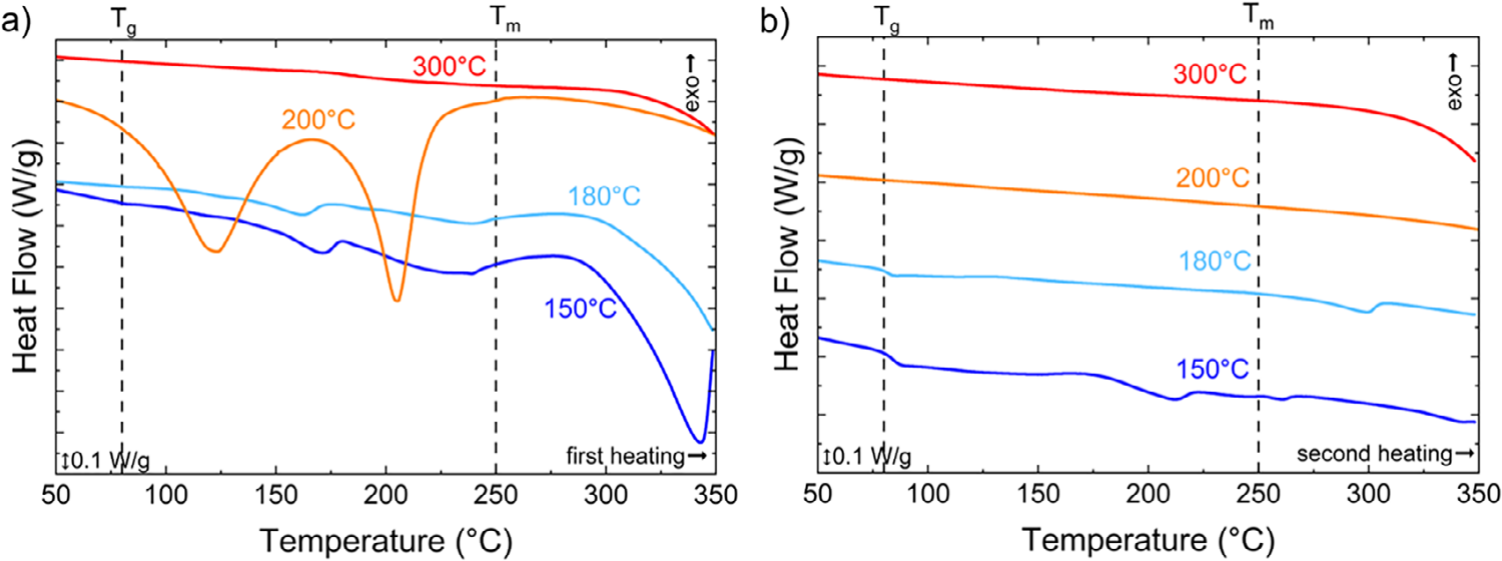
DSC thermograms of dried products from hydrolysis of ReCryst_vPET on a) first and b) second heating cycles at 10°C min^−1^. The temperature for the hydrolysis reaction is noted next to the thermogram curves on the plot. The dashed vertical lines are associated with T_g_ and T_m_ of bulk PET.

The DLS data demonstrates the presence of particles within the solutions after hydrolysis, but it is not clear that these are microplastics. As the chemistry of the hydrolysis products and the PET is similar, most spectroscopic techniques are limited in their ability to identify PET from the high concentration of TPA in the product. Separations to selectively remove the TPA and oligomers are tedious and prone to loss of the microplastics in filters or introduction of microplastics through contamination [58]. However, the thermal properties of TPA and PET differ substantially due to their differences in crystal structure as shown in Figures S1 and S5. Figure 4 illustrates the DSC thermograms from the dried solids after hydrolysis, excluding any visible macroscopic PET that was remaining after the hydrolysis. The heat flow from the initial heating of these solids recovered from hydrolysis is shown in Figure 4a. Solids from hydrolysis at 150°C exhibit a small shift in the heat capacity near T_g_ of PET. T_g_ is a signature of the amorphous glass in PET, so a limited signature of a glass is expected if the amorphous segments are preferentially hydrolyzed. For the products from hydrolysis at 150°C and 180°C, there are two melting peaks: one near 160–170°C and the other near 230°C on the first heating. These transitions are below the melting point initially measured for the PET. Prior reports examining the depolymerization products from glycolysis of PET have reported an equilibrium betweenBHET and the dimer of BHET, where both forward and reverse reactions are occurring [59]; a melting peak near 165°C was attributed to the crystalline dimer of BHET [59, 60]. The lower temperature endotherm for the hydrolysis products is likely from the dimer of BHET. Examination of Figure 4a illustrates a slight shift in this first melting transition between the products from hydrolysis at 150°C and 180°C – this shift is likely associated with a slight change in the composition (co‐crystallization) in the crystal [60].

The endotherm at higher temperature for the 180 and 150 °C hydrolysis products is broad and lacks clear multiple peaks as would be expected for discrete molecules [60]. The melting point for polymer crystals depends both on their size and local environment [61]. Based on the hypothesized mechanism shown in Figure 1, the hydrolysis reactions will occur around the crystallites to alter the polymer chemistry near to the crystal surface. For example, ionomers of polyethylene with only a few percent inclusion of ionic functionality on the vinyl backbone exhibit a melting point nearly 15°C less than pure polyethylene [62]. Prior work on PET nanoparticles demonstrated a broad, reduced melting point near 200°C [35], which is consistent with the data shown in Figure 4a. The endotherm for subliming/melting TPA begins near 300°C. The overall enthalpy for melting of the PET relative to the dimer is decreased after hydrolysis at 180°C relative to 150°C, suggesting fewer PET NPs are present after hydrolysis at 180°C. Hydrolysis at higher temperatures results in products with significantly altered thermograms. The residual after hydrolysis at 200°C shows well‐defined endotherms with minima in the heat flow near 120°C and 200°C. These could be associated with BHET and its dimer based on the starting temperature of the endotherms. However, there are no signatures for PET from either shifts in heat flow near T_g_ or a broad melting endotherm. Products from hydrolysis at 300°C exhibit no apparent crystalline phases below the sublimation/melting temperature of TPA. This is consistent with prior reports on the hydrolysis of PET that high yields of TPA are obtained at temperatures greater than its melting point [28].

A second heating cycle was used to further understand the products from the hydrolysis reaction. The DSC thermograms shown in Figure 4b illustrate that a clear T_g_ for PET is visible on reheating for the samples hydrolyzed at 150°C and 180°C. This is consistent with the interpretation of the broad melting endotherm from 200°C to 270°C in the initial heating (Figure 4a), although there is potential for re‐polymerization of PET during the DSC measurement. The endotherms attributed to the BHET dimer are absent on re‐heating, indicating loss of this species on the initial heating to 350°C or its inability to crystallize in the mixture present. Interestingly, the endotherm for the PET melting in the sample from hydrolysis at 150°C is shifted to a lower temperature between 175–225°C, which is similar to that reported by Kumar and coworkers for PET microplastics formed from hydrolysis [35]. A weak endotherm near 260°C also emerges; a higher melting temperature has previously been reported for blends of linear and branched PET, which could explain the melting at a temperature exceeding the original PET [63].

Both endotherms are missing from the DSC thermograms for samples depolymerized at higher temperatures on the second heating. For the products of hydrolysis at 180°C, the peak near 300°C is consistent with BHET/TPA melting/sublimation. This peak is relatively weak, which suggests most of the TPA/BHET was removed in the first heating. Loss of the endotherm within the melting region on re‐heating suggests that the particles formed in the solution from hydrolysis at 180°C may not be associated with polymeric PET and instead oligomers formed from hydrolysis of PET at the crystal surfaces. However, there is an apparent T_g_ for PET present in the hydrolysis products from reaction at 180°C. The thermograms of the hydrolysis products from reactions at 200°C and 300°C are essentially featureless, indicative of no residual or reformed PET in the sample. As all reactions are performed at lower temperatures than the normal melting point of PET, except the hydrolysis at 300°C, the reason for the difference in resilience of the PET in hydrolysis must be related to something other than simply the melting point of the PET. The dynamics of the polymer segments near the crystal surfaces, termed rigid amorphous fraction (RAF), differ from the bulk amorphous regions in PET [53]. This change in dynamics may also impact reactivity and accessibility of water. Hydrolysis reactions in the RAF may be suppressed only at temperatures much below the melting point; this would explain why there appears to be significantly more PET NPs from hydrolysis at 150°C than at the other temperatures examined.

**FIGURE 5:**
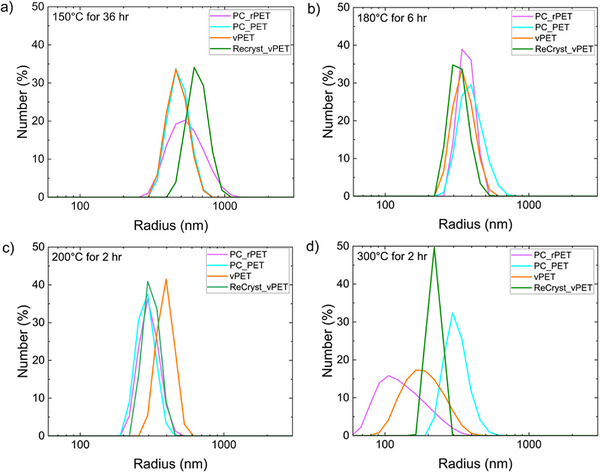
Particle size distribution determined from DLS for the four different PET sources after hydrolysis at (a) 150°C, (b) 180°C, (c) 200°C, and (d) 300°C. The solids from hydrolysis were diluted with DMSO at 9:1 DMSO: water for the light scattering measurements.

The generality of these results was investigated using PET with significant differences in their crystallinity and sourcing origins (Table 1). Figure 5 illustrates how the distribution of particles obtained under different hydrolysis conditions is impacted by the sourcing of the PET. The associated autocorrelation functions and additional particle distributions obtained from the hydrolysis of other PET sources are shown in Figures S6 and S7, respectively. From hydrolysis at 150°C, some differences in the particle size are observed with the PC_PET and vPET exhibiting similar sizes (Figure 5a). Both of these PET samples are initially clear and transparent; thus, they would be expected to have the smallest crystal sizes and generate the smallest NPs based on a crystal release mechanism (Figure 1). On recrystallization of the vPET, the crystallinity of the sample grows significantly and likely is associated with larger crystal sizes, which agrees with the particle sizes generated. The PC_rPET is more crystalline than the bottle produced by non‐recycled PET. The particles generated exhibit a broad distribution that spans the range of the other samples. This breadth is likely associated with some heterogeneities in the recycled PET that produce local differences in the crystallization of the PET.

Hydrolysis of these different PET samples at 180°C (Figure 5b) and 200°C (Figure 5c) shows limited differences in the sizes of the particles produced. Both temperatures are well below the observed melting point (Figure S1) for the PET examined here. This lack of sensitivity to the initial state of the crystals in the PET suggests that these conditions do not produce appreciable microplastics through the mechanism illustrated in Figure 1 and previously reported for PET microplastics [35]. Hydrolysis at 300°C appears to produce greater variance in the particle sizes, but these are all similar or less than the sizes obtained from hydrolysis at 200°C. These differences are likely associated with the products as additional reaction pathways are present that can change the product distribution [30].

**FIGURE 6:**
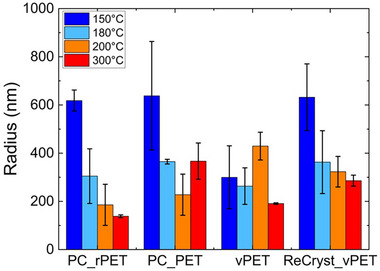
Role of reaction temperature on the particle sizes observed in DLS for the four sources of PET examined.

Figure 6 quantifies these differences in the particle sizes for the different sources of PET. There is a general trend that the size obtained from reactions at 150°C is generally significantly larger than that at higher reaction temperatures. Moreover, in many cases, the size observed at these higher reaction temperatures is consistent with control measurements of TPA in DMSO: water, where nearly 200 nm particles were found (Figure S2). The only outlier is the vPET, where the particle size is not follow this trend, with the particle size from hydrolysis at 150°C matching the TPA control. This could be associated with this PET beginning with a low crystallinity and likely smaller crystals. Low crystallinity would lead to a lower potential for NPs from the release of the crystalline domains (see schematic in Figure 1). Lower concentration of NPs from less crystals present would skew the particle size toward the TPA assemblies in solution. Additionally, vPET is transparent initially, which also indicates that the crystal domains within the vPET will be small. Smaller crystals would reduce the melting point for the PET, which could potentially shift below 150°C below after hydrolysis of amorphous regions and some tie chains. The general consistency in the particle size from the hydrolysis at 150°C suggests that this reaction condition leads to the formation of some microplastics in the products, except potentially the least crystalline sample examined.

These data suggest that the crystals in PET do not fully suppress the depolymerization during hydrolysis at temperatures less than T_m_. Hydrolysis reactions at temperatures exceeding 150°C appear to sufficiently rapidly depolymerize the crystalline PET to prevent appreciable NPs in the products, but some microplastics may be formed at 180°C for the most crystalline PET sample. The reactivity of the crystalline PET is likely to be influenced by solvent selection for solvolysis and any catalysts, but the potential formation of microplastics during low temperature PET chemical recycling should be assessed to avoid unintended production of micro‐and nano‐plastics during recycling.

## 3 Conclusions

Recent work examining the origins of micro‐ and nano‐plastic formation suggests that microplastics are generated through the preferential degradation of amorphous regions in semicrystalline polymers [35]. Sustainability metrics pushing recycling processes to reduce energy use may inadvertently generate microplastics in semicrystalline polymers when the reaction temperature is less than the melting point. Here, we examined the potential for microplastic generation with the hydrolysis of PET. Hydrolysis temperatures from 150 to 300°C were examined. The simple identification of nanoplastics through dynamic light scattering was challenged by the aggregation of the hydrolysis product, terephthalic acid (TPA), in mixtures of DMSO – water. Products diluted visually to clear by the addition of DMSO remained to show particles of the order of hundred nm, consistent with TPA in DMSO‐water mixtures. Through additional measurements of the thermal transitions using differential scanning calorimetry (DSC), nanoplastics of PET were identified in the products from hydrolysis at 150°C after 36 h of reaction. Hydrolysis at 180°C leads to products with similar thermograms, but the particle size is significantly reduced and is statistically similar to the particles of TPA alone. No clear signatures of microplastics were observed in the thermograms of the products from reactions at higher temperatures. These results demonstrate that nanoplastics can be formed during the hydrolysis of PET at low temperature, and these particles can be stable in the product solution even at long reaction times. Future work will focus on understanding if nanoplastics are generated under catalytic conditions or other solvolysis reactions for the depolymerization of PET.

## Author Contributions

S.F.Y. contributed to the investigation, formal analysis, visualization, and writing of the original draft. P.M.G. contributed to the investigation and methodology. P.P. contributed to the methodology and investigation. P.E.S. contributed to resources, funding acquisition, supervision, and writing through review and editing. B.D.V. contributed to conceptualization, supervision, project administration, and writing of both the original draft and the review and editing.

## Conflicts of Interest

There are no conflicts to declare.

## Supporting information




**Supporting File**: marc70222‐sup‐0001‐SuppMat.pdf.

## Data Availability

The data that support the findings of this study are available in the supplementary material of this article.
