# Supplementary material for: Nanoplastics in Depolymerization Products from Hydrolysis of Poly(Ethylene Terephthalate) in the Solid State
Source: Macromol Rapid Commun. 2026 Feb 6;47(8):e00776. doi: 10.1002/marc.202500776 (PMC13087854; doi:10.1002/marc.202500776)
Supplement: Supplementary file 1 — Supporting File: marc70222‐sup‐0001‐SuppMat.pdf. [file MARC-47-e00776-s001.pdf]

SUPPLEMENTARY INFORMATION

Nanoplastics in Depolymerization Products from Hydrolysis of Poly(ethylene terephthalate) in the Solid State

Sierra F. Yost, Peter M. Guirguis, Patricia Pereira, Philip E. Savage, and Bryan D. Vogt*

Robert V. Waltemeyer Department of Chemical Engineering, The Pennsylvania State University, University Park, PA 16802

*To whom correspondence should be addressed: bdv5051@psu.edu (B.D.V)

Contents

Experimental Details

Figure S1. DSC thermograms for individual PET sources on a) first heating and b) cooling at 10 °C min^{-1}.
Figure S2. Hydrodynamic radii of hydrolysis products from ReCryst_vPET when dissolved in a) 75:25 and b) 9:1 (v:v) DMSO:water from multiple samples for statistical analysis.
Figure S3. Autocorrelation function for ReCryst_vPET products from hydrolysis at a) 150 °C for 36 h, b) 180 °C for 6 h, c) 200 °C for 2 h and d) 300 °C for 2 h.
Figure S4. a) Hydrodynamic radius distribution on number average basis and b) autocorrelation function of TPA (7.5 mg/mL) in a 9:1 and 75:25 v:v of DMSO and water. One sample of TPA in 9:1 solution did not exhibit any appreciable correlated scattering on the time scales measured.
Figure S5. DSC thermogram of pure TPA on first heating (black line) and cooling (red line) at 10 °C min^{-1}.
Figure S6. DLS autocorrelation functions for hydrolysis products of PC_rPET, PC_PET, and vPET at a) 150 °C for 36 h, b) 180 °C for 6 h, c) 200 °C for 2 and d) 300 °C for 2 h.
Figure S7. Additional DLS data from 3 PET sources for products from hydrolysis at a) 150 °C for 36 h, b) 180 °C for 6 h, c) 200 °C for 2 h and d) 300 °C for 2 h.

Experimental

Materials and methods

Dimethylsulfoxide (99.8+% HPLC grade, DMSO) was purchased from Thermo Scientific. Water was purified using reverse osmosis, ion exchange and high-capacity ion exchange, UV sterilization, and submicron filtration units. PET was sourced from a variety of producers to examine the influence of the prior history of the PET on microplastic formation. Terephthalic acid (TPA) was purchased from TCI (T0166). Two different post-consumer PET samples were examined from Diet Coke bottles: a 16.9 oz bottle without recycled content labeling (PC_PET) and a 20 oz bottle labeled as 100% recycled material (PC_rPET). Samples were cut from the same location (underneath the label, not including the adhesive) on the bottle into approximately 2 cm x 2 cm pieces. 2 mm thick PET sheets (300 mm x 300 mm) were purchased from Sigma Aldrich (vPET, GF14511215). The sheets were cut into square coupons (approximately 2 cm x 2 cm) with scissors for hydrolysis reactions. The crystallinity of the vPET sheet was increased (ReCryst_vPET) through melting and re-crystallization in a Carver press at 5000 psi by heating to 280 °C for 10 min and subsequent slow cooling simply from the thermal mass of the platens of the press by switching off the power to the heat. The ReCryst_vPET was removed from the press after fully cooled to room temperature.

Hydrolysis of the PET coupons (~350 mg) was performed in closed, stainless steel Swagelok reactors with an internal volume of ~ 4 mL, following the method for isothermal hydrolysis of PET described by Pereira, et al.[28] The reactor was loaded with 3.5 mL of deionized water and one PET coupon and heated in an isothermal Techne fluidized sand bath at 150-300 °C for 2-36 h. Subcritical water has been found to be the most effective at depolymerization of PET via hydrolysis.[28, 51] A two and one half minute temperature ramp was employed to increase the reactor temperature from room temperature to the desired set temperature and this ramp is included in the reported reaction times.[52] After the desired reaction time, the reactors were removed from the sand bath and immediately immersed in room temperature water to quench the reaction. Any gaseous products from the reaction were not collected on opening the vessel at room temperature. The product from the reactor was diluted with DMSO at either 75:25 or 90:10 (v:v) DMSO:water. These compositions led to clear solutions from originally white, turbid, phase separated water-TPA mixtures in less than one min. The residual macroscopic PET after hydrolysis was allowed to sediment in the DMSO:water solution and removed.

Characterization

Differential scanning calorimetry (DSC). Thermograms of PET were obtained using a TA Instruments DSC 250 under an N_2 flow of 50 mL min^{-1} (<2 ppm moisture, Linde). DSC was

used to quantify thermal transitions, crystallization and glass transition, of the materials before hydrolysis and the recovered solids after hydrolysis without inclusion of any macroscopic PET in the product. The recovered solids tested used approximately 1 mL of suspended solids in the TPA rich phase. The solution was taken from the middle of the vial to avoid removal of PET solids from the bottom of the vial. The solids were dried under ambient conditions to evaporate most of the water present. Approximately 2 mg of solids from hydrolysis products were sealed in Tzero aluminum pans. The DSC thermograms were measured between 30 °C and 350 °C with heating and cooling at 10 °C min^{-1} for two thermal cycles. The melting temperature (T_m), crystallization temperature (T_c), glass transition temperature (T_g) and enthalpy of melting (H_m) on the first heating and cooling cycles were determined using TRIOS software. T_g was calculated using the mid-point method. The percent crystallinity (X_c) of the original PET samples was determined from the measured H_m relative to the known crystallization enthalpy of PET (140.1 J/g).[53] Table 1 summarizes these characteristics for the different PET samples examined in this work.

Dynamic light scattering (DLS). Visually clear solutions in DMSO:water were characterized by DLS. Light scattering was performed at 25 °C using a Zetasizer Nano ZS with 633 nm He-Ne laser for the light source and auto-attenuation functionality. The detector angle was 173° for non-invasive back scatter (NIBS). The scattered intensity was measured for 200 s for each measurement. Each sample was measured in triplicate. The autocorrelation function from the time dependent intensity was fit to cumulant and size-distribution models with the Zetasizer Software.

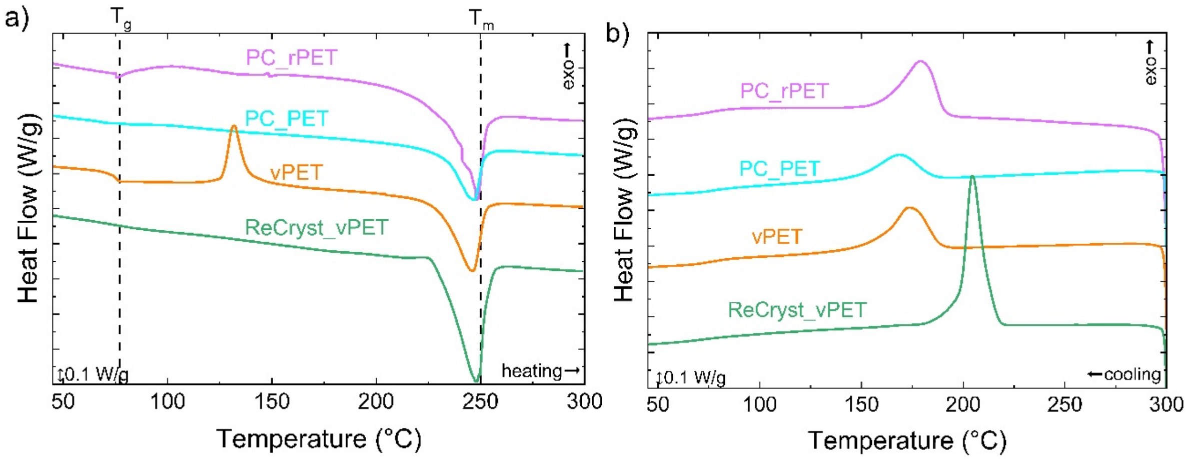

Figure S1. DSC thermograms for individual PET sources on a) first heating and b) cooling at 10 °C min^{-1}.

Dynamic Light Scattering (DLS) Data Acquisition

The viscosity of the 9:1 and 75:25 (v:v) solutions of DMSO: water were 2.7 cP and 3.68 cP, respectively.[1] These values were used for the calculation of the particle size distributions.

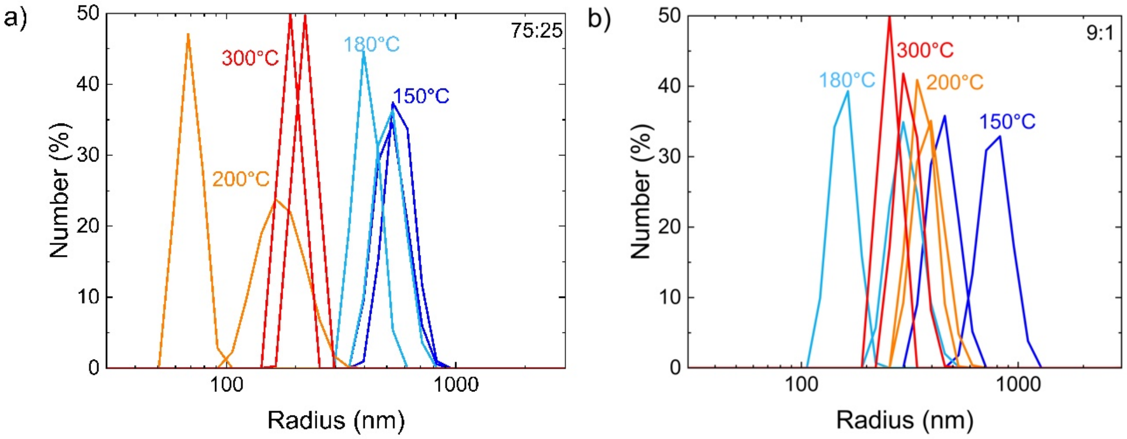

Figure S2. Hydrodynamic radii of hydrolysis products from ReCryst_vPET when dissolved in a) 75:25 and b) 9:1 (v:v) DMSO:water from multiple samples for statistical analysis.

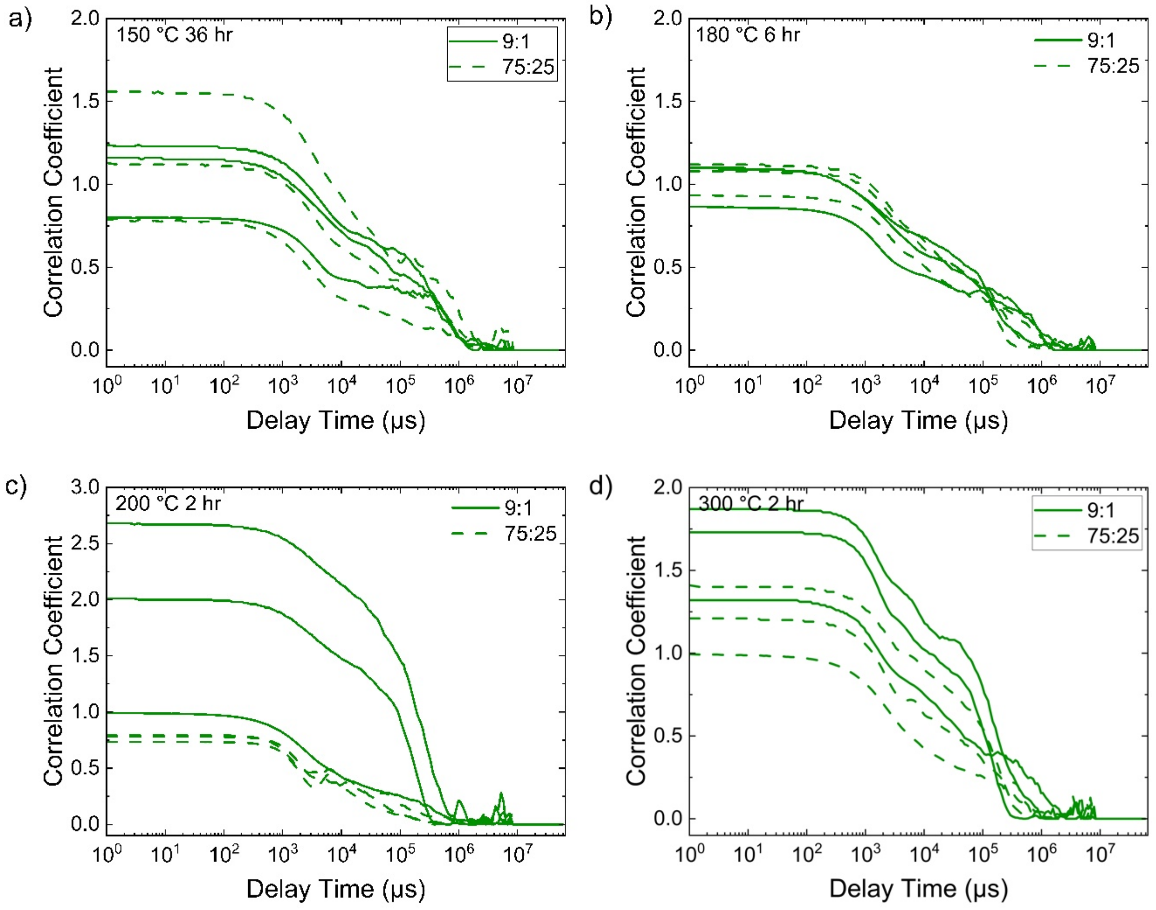

Figure S3. Autocorrelation function for ReCryst_vPET products from hydrolysis at a) 150 °C for 36 h, b) 180 °C for 6 h, c) 200 °C for 2 h and d) 300 °C for 2 h.

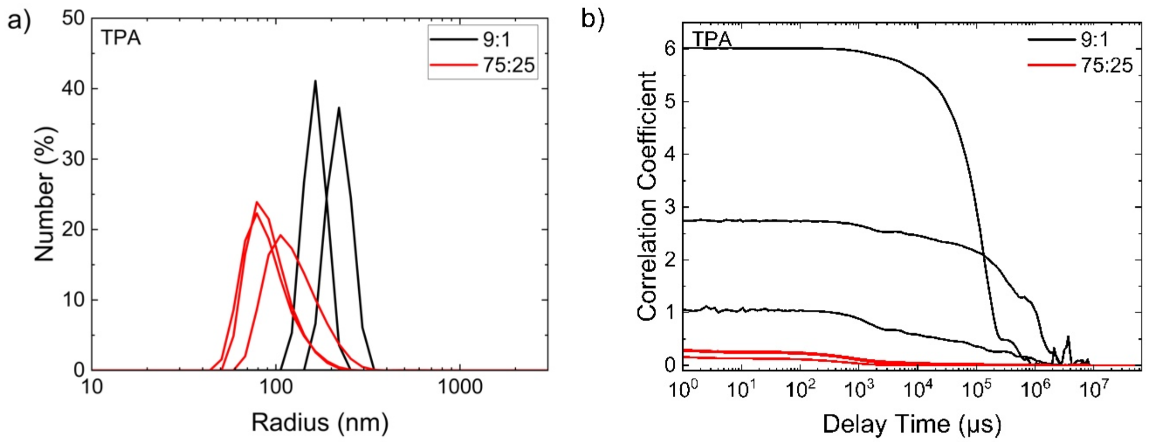

Figure S4. a) Hydrodynamic radius distribution on number average basis and b) autocorrelation function of TPA (7.5 mg/mL) in a 9:1 and 75:25 v:v of DMSO and water. One sample of TPA in 9:1 solution did not exhibit any appreciable correlated scattering on the time scales measured.

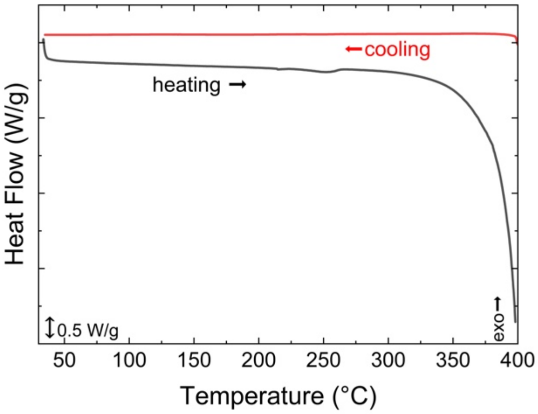

Figure S5. DSC thermogram of pure TPA on first heating (black line) and cooling (red line) at 10 °C min^{-1}.

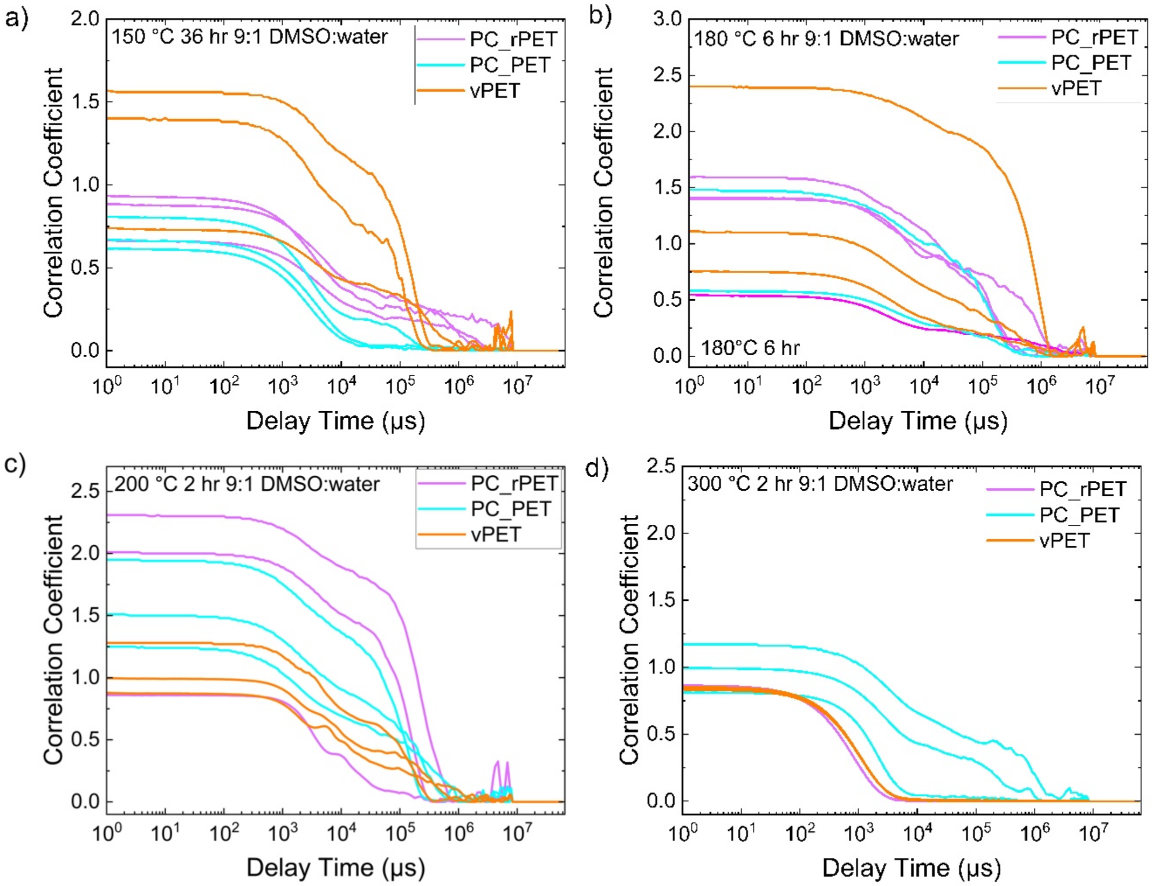

Figure S6. DLS autocorrelation functions for hydrolysis products of PC_rPET, PC_PET, and vPET at a) 150 °C for 36 h, b) 180 °C for 6 h, c) 200 °C for 2 h and d) 300 °C for 2 h.

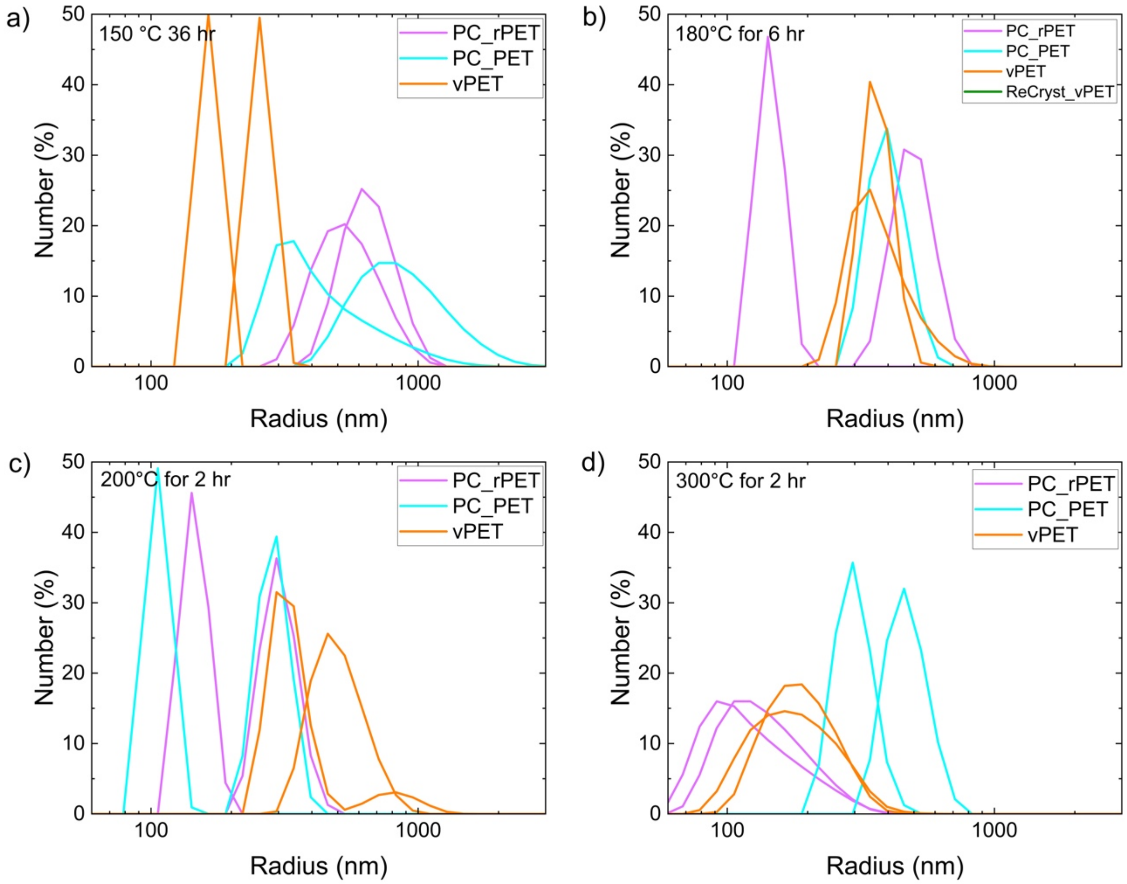

Figure S7. Additional DLS data for products from hydrolysis at a) 150 °C for 36 h, b) 180 °C for 6 h, c) 200 °C for 2 h and d) 300 °C for 2 h from 3 PET sources.

References

[1] R. G. LeBel and D. A. I. Goring, 1962, **7**, 100-101
